# Suppressive Effects of Transient Receptor Potential Melastatin 8 Agonist on Epileptiform Discharges and Epileptic Seizures

**DOI:** 10.3389/fphar.2021.766782

**Published:** 2021-10-01

**Authors:** Hiroshi Moriyama, Sadahiro Nomura, Hirochika Imoto, Takao Inoue, Yuichi Fujiyama, Kohei Haji, Yuichi Maruta, Hideyuki Ishihara, Michiyasu Suzuki

**Affiliations:** ^1^ Departments of Neurosurgery, Graduate School of Medicine, Yamaguchi University, Ube, Japan; ^2^ Epilepsy Center, Yamaguchi University Hospital, Ube, Japan

**Keywords:** electrocorticograms, epileptiform discharges, focal epilepsy, penicillin G potassium, seizure score, transient receptor potential melastatin 8 knockout mice

## Abstract

Epilepsy is a relatively common condition, but more than 30% of patients have refractory epilepsy that is inadequately controlled by or is resistant to multiple drug treatments. Thus, new antiepileptic drugs based on newly identified mechanisms are required. A previous report revealed the suppressive effects of transient receptor potential melastatin 8 (TRPM8) activation on penicillin G-induced epileptiform discharges (EDs). However, it is unclear whether TRPM8 agonists suppress epileptic seizures or affect EDs or epileptic seizures in *TRPM8* knockout (TRPM8KO) mice. We investigated the effects of TRPM8 agonist and lack of TRPM8 channels on EDs and epileptic seizures. Mice were injected with TRPM8 agonist 90 min after or 30 min before epilepsy-inducer injection, and electrocorticograms (ECoGs) were recorded under anesthesia, while behavior was monitored when awake. TRPM8 agonist suppressed EDs and epileptic seizures in wildtype (WT) mice, but not in TRPM8KO mice. In addition, TRPM8KO mice had a shorter firing latency of EDs, and EDs and epileptic seizures were deteriorated by the epilepsy inducer compared with those in WT mice, with the EDs being more easily propagated to the contralateral side. These findings suggest that TRPM8 activation in epileptic regions has anti-epileptic effects.

## Introduction

Epilepsy is the most common central nervous system disease. Accounting for approximately 30% of epilepsy cases, intractable epilepsy involves inadequate control of seizures and is rarely improved even when multiple antiepileptic drugs are administered or therapeutic agents are changed (Kwan and Brodie, 2000; Golyala and Kwan, 2017; Sheng et al., 2018). Thus, the development of antiepileptic drugs based on newly identified mechanisms is predicted.

Transient receptor potential melastatin 8 (TRPM8) activation suppresses drug-induced epileptiform discharges (EDs) (Moriyama et al., 2019), and the anti-epileptic-like effects of TRPM8 agonists have received attention. TRPM8 channels are expressed in small numbers in the rodent somatosensory cortex (Ordas et al., 2019) and are activated by TRPM8-selective agonists (Behrendt et al., 2004) or cold temperatures between 10 and 26°C (Bautista et al., 2007). EDs in patients with refractory epilepsy are suppressed when the epileptic focus is cooled to 15°C, which is the temperature that activates TRPM8 channels (Nomura et al., 2014; Nomura et al., 2017). Taken together, these reports suggest that TRPM8 activation induced anti-epileptic effects. However, the effects of TRPM8 activation on epileptic seizures remain unknown.

Penicillin G potassium (PG), an epilepsy-inducer, inhibits GABA_A_ receptor (Tsuda et al., 1994; Sugimoto et al., 2002), leading to EDs and epileptic seizures (Kida, 2012; Moriyama et al., 2019; Fujii et al., 2012). TRPM8 activation by icilin, a TRPM8 and transient receptor potential ankyrin 1 (TRPA1) agonist, suppresses EDs (Moriyama et al., 2019), indicating that a lack of TRPM8 channels exacerbates epileptic seizures. However, the effects of no TRPM8 channels on EDs and epileptic seizures is unclear.

It is important to suppress the development of EDs because epileptic seizures are induced by excessive synchronized excitement of nerve cell groups. Nonetheless, the effects of TRPM8 agonists on the development of EDs remains to be established.

In this study, we aimed to clarify the effects of TRPM8 agonist and the absence of TRPM8 channels on EDs, epileptic seizures, and the development of EDs.

## Materials and Methods

### Animals

Male C57BL/6N mice aged 9–11 weeks, weighing 24–30 g (Kyudo, Saga, Japan), and *TRPM8* homozygous knockout (TRPM8KO) mice (Prof. Makoto Tominaga, Thermal Biology Group, Exploratory Research Center on Life and Living Systems, Okazaki, Japan) were housed in groups of five mice per cage and kept under standard laboratory conditions in a temperature- and humidity-controlled room (25 ± 2°C and 55 ± 5%, respectively) on a 12-h light/dark cycle (lights on at 8:00 a.m.) (Dhaka et al., 2007). The animals had free access to food and water. The animal care and experimental procedures were approved by the Experimental Animal Care and Use Committee of Yamaguchi University School of Medicine, Japan. All experiments were performed in accordance with the guidelines of the Japan Association for Laboratory Animal Facilities of National University Corporations.

### Drugs

Pentylenetetrazol (PTZ; Tokyo Chemical Industry, Japan) and penicillin G potassium (PG: Meiji, Tokyo, Japan) were dissolved in saline. WS-3 (Funakoshi, Japan) was dissolved in 1% dimethyl sulfoxide (DMSO; Merck KGaA) in saline.

### Drug-Induced Epileptic Seizure Model

To determine whether TRPM8 channel deficiency exacerbates generalized and focal seizures in mice, we compared PTZ- and PG-induced seizures in WT and TRPM8KO mice. PTZ-induced seizure mice were generated according to a previously reported method (Mizoguchi et al., 2011). Mice were intraperitoneally (i.p.) administered a single dose of PTZ (40 mg/kg). A model with focal epilepsy originating in the left cerebral cortex was generated by modifying a previously reported method (Moriyama et al., 2019). The animals were anesthetized with urethane (1.75 g/kg i.p.) or through inhalation of sevoflurane (Pfizer Japan, Tokyo, Japan) (3% for induction, 1.5% for maintenance) and immobilized using a stereotaxic apparatus. To maintain the body and brain temperature in each rat, rectal temperature was adjusted to 37.5 ± 0.5°C using a heat pad. We created three burr holes measuring 1.0 mm in diameter, two for the ECoG recording and the other for the reference electrode, above the bilateral sensorimotor cortices and cerebellum using the following coordinates: 1.0 mm posterior; ± 2.0 mm lateral from the bregma, and 2.0 mm posterior from the lambda. We also placed a thin thermocouple (IT-24, Physitemp, Tokyo, Japan), and two ECoG and reference electrodes in the bilateral somatosensory cortices and cerebellar area between the skull and dura. We placed a ground electrode in the tail. A small slit in the dura was created to insert an injection cannula (0.4 mm diameter × 40 mm length, and 3 µl volume; EIM-40, Eicom, Kyoto, Japan). The injection cannula was inserted to a depth of 0.75 mm from the brain surface. PG at a concentration of 200 IU/µl was injected intracortically for 10 min at a rate of 0.1 µl/min using a 10 µl Hamilton syringe and a microinjection pump (ESP-64, Eicom, Japan).

### Drug Treatments

WS-3 was administered intracortically for 10 min, with WS-3 administration starting 90 min after the PG injection at a rate of 0.1 µl/min using a microinjection pump. In the prevention of epileptic seizures induced by TRPM8 activation, DMSO or WS-3 was administered to mice 30 min before PG injection.

### Seizure Score

**FIGURE 1:**
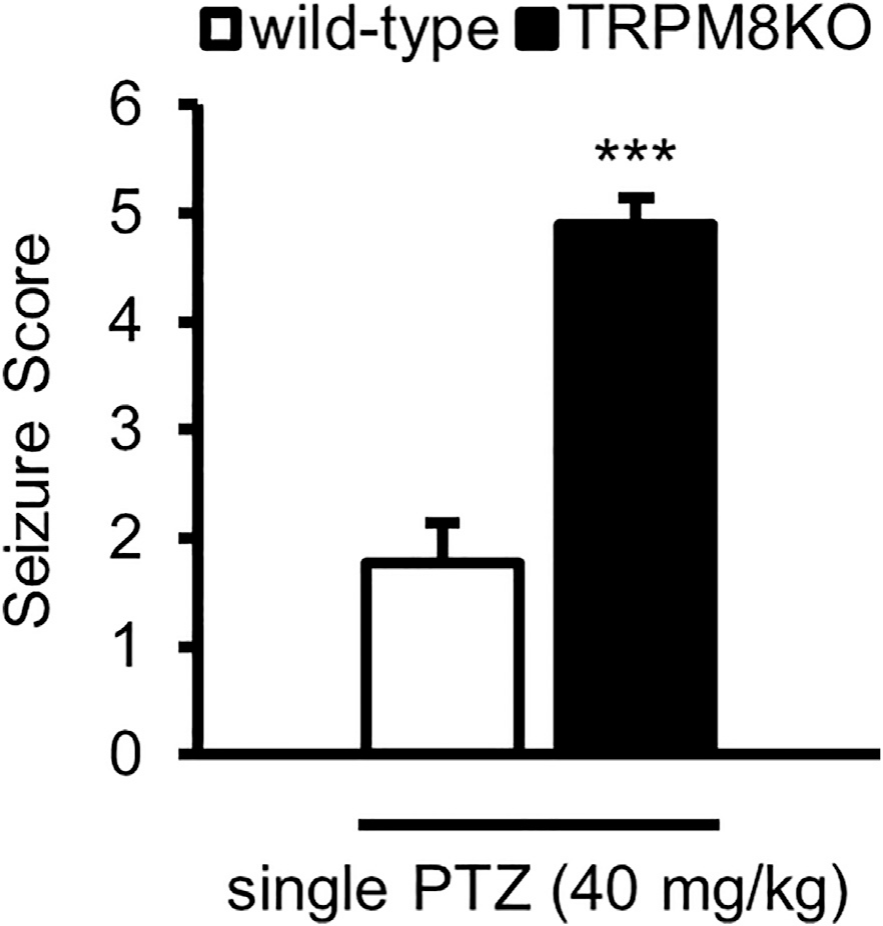
Exacerbation of seizure score by lack of TRPM8 channels. Mean seizure scores for WT (white: *n* = 6) and TRPM8KO (black: *n* = 6) mice, which were at the maximum during 60 min. The results are shown as mean ± SEM; ***p < 0.001, Student’s *t*-test. TRPM8, transient receptor potential melastatin 8; PTZ, pentylenetetrazol; WT, wild type; SEM, standard error of the mean.

**FIGURE 2:**
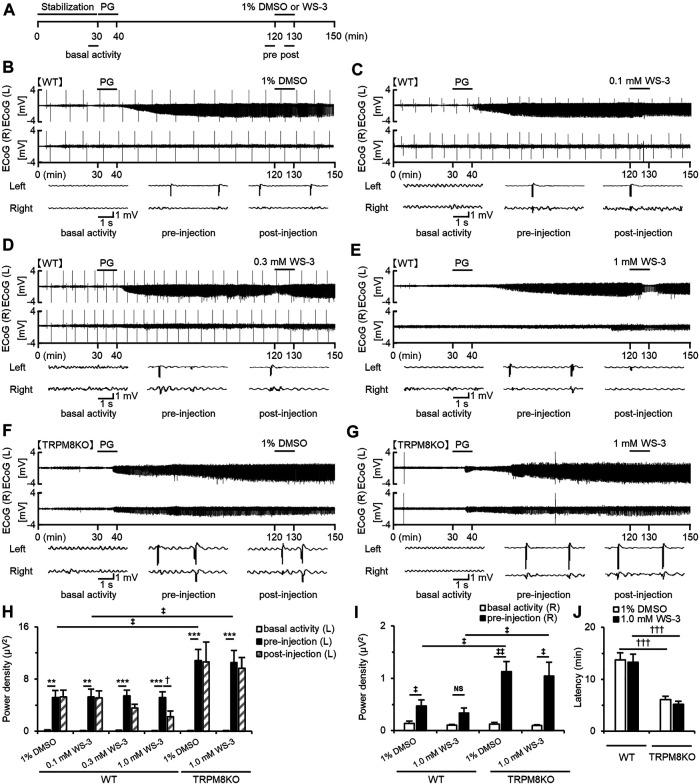
Suppressive effects of WS-3 on PG-induced EDs in WT mice and deterioration of EDs due to lack of TRPM8 channels. **(A)** Experimental protocol. Mice were anesthetized with urethane. The power of the low beta band in ECoG was integrated for 5 min during the basal activity (25–30 min) and the pre- (115–120 min) and post- (125–130 min) injection periods. Examples of changes in EDs with **(B)** PG + 1% DMSO (*n* = 6), **(C)** PG + 0.1 mM WS-3 (*n* = 5), **(D)** PG + 0.3 mM WS-3 (*n* = 6), and **(E)** PG + 1.0 mM WS-3 (*n* = 6) in WT mice, and **(F)** PG + 1% DMSO (*n* = 6) and **(G)** PG + 1.0 mM WS-3 (*n* = 6) in TRPM8KO mice. **(B–G)** Representative ECoG traces for 5 s during basal activity and the pre- and post-injection period. **(H)** The integrated value of the low beta bands in the affected (Light) side during basal activity (white) and the pre- (black) and post-injection (grey-oblique line) periods. **(I)** The integrated value of the low beta bands in the contralateral (Right) side during basal activity (white) and the pre-injection (black) period. **(J)** Latency of EDs evoked in WT (white) and TRPM8KO mice (grey). The results are shown as mean ± SEM; **p < 0.01, ***p < 0.001, Tukey’s test; ^†^ *p* < 0.05, ^†††^ *p* < 0.001, Student’s *t*-test; ^‡^ *p* < 0.05, ^‡‡^ *p* < 0.01, Welch’s *t*-test. NS, no significant.

**FIGURE 3:**
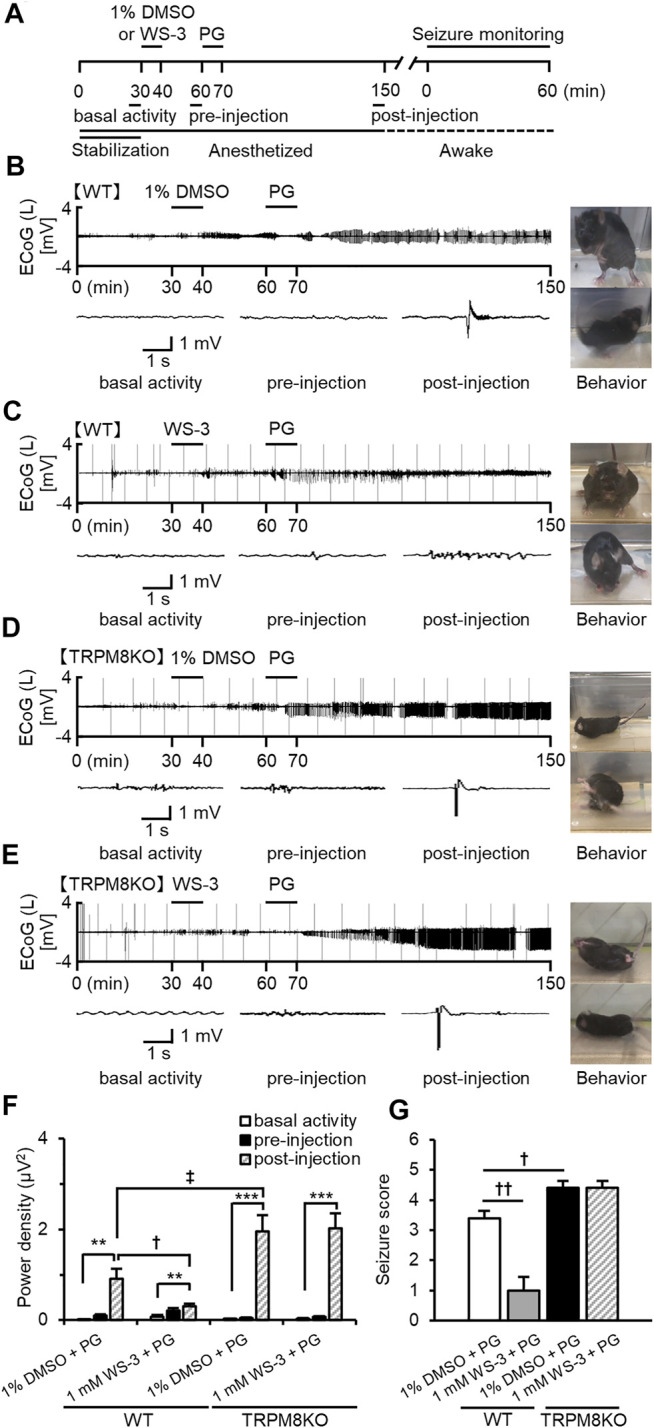
Suppressive effects of WS-3 on ED development and epileptic seizures in WT mice, and deterioration of ED development and epileptic seizures resulting from lack of TRPM8 channels. **(A)** Experimental protocol. Mice were anesthetized with sevoflurane. The power of the low beta band in ECoGs was integrated for 5 min during basal activity (25–30 min) and the pre- (55–60 min) and post- (145–150 min) injection periods. Examples of changes in EDs with **(B)** PG + 1% DMSO (*n* = 5), **(C)** PG + 1.0 mM WS-3 (*n* = 6) in WT mice, and (D) PG + 1% DMSO (*n* = 5), and **(E)** PG + 1.0 mM WS-3 (*n* = 5) in TRPM8KO mice. **(B–E)** Representative ECoG traces for 5 s during basal activity and the pre- and post-injection periods. **(B–E)** Typical behavior during seizure monitoring. **(F)** The integrated value of the low beta band in the contralateral side during basal activity (white) and the pre-injection (black) period. **(G)** Mean seizure scores that were at the maximum during the 60-min period for vehicle (white) or WS-3 (grey) administration in WT mice and for vehicle (black) or WS-3 (grey-oblique line) administration in TRPM8KO (black) mice. The results are shown as mean ± SEM; **p < 0.01, ***p < 0.001, followed by Tukey’s test; ^†^ *p* < 0.05, ^††^ *p* < 0.01, Student’s *t*-test; ^‡^ *p* < 0.05, Welch’s *t*-test.

The behavior of mice was observed for 1 h following PTZ injection (Figure 1). Because anesthesia was continuously maintained for at least 5 h by urethane, we did not perform seizure monitoring (Figure 2A). The behavior of mice was observed for 1 h after the end of ECoG recording for 2.5 h (Figure 3A). Referring to previous studies (Mizoguchi et al., 2011), PTZ-, or PG-induced seizure intensity was scored as follows: stage 0, no response; stage 1, ear and facial twitching; stage 2, convulsive twitching axially through the body; stage 3, myoclonic jerks and rearing; stage 4, turning over onto the side, wild running, and wild jumping; stage 5, generalized tonic-clonic seizures; and stage 6, death.

### ECoG Recording

ECoG was recorded by modifying a previously reported method (Moriyama et al., 2019). EDs developed 10–20 min after PG injection and reached a steady state 1.5 h after PG injection (Moriyama et al., 2019), remaining stable for at least 5 h (data not shown). Because icilin, a TRPA1 and TRPM8 agonist, eliminated all EDs for only a few minutes (Moriyama et al., 2019), we regarded the drug efficacy period as 5 min from 5 to 10 min after the start of TRPM8 agonist injection. ECoGs were continuously recorded for 2.5 h (0.5 h for ECoG stabilization and 1.5 h for stabilization of PG-induced EDs, and 0.5 h for drug efficacy evaluation; Figure 2A; and 0.5 h for ECoG stabilization and 0.5 h for ECoG stabilization after 1% DMSO or WS-3 injection, and 1.5 h for stabilization of PG-induced EDs; Figure 3A).

ECoGs were amplified by a bio-amplifier (EX-1; Dagan Corporation, Minneapolis, MN) using an analogue-to-digital converter at a sampling rate of 2 kHz (PowerLab 8/30; AD Instruments, Castle Hill, Australia). The conditions for recording ECoGs were as follows: low-frequency filter, 0.1 Hz; high-frequency filter, 10 kHz; and notch filter, off.

To evaluate the effects of TRPM8 channel activation on PG-induced EDs, the left ECoG was fast Fourier-transformed. We calculated the power of the low beta band (14–24 Hz) during the subsequent 5 min periods using Lab Chart Pro v. 8.1.16 (AD Instruments), as follows: basal activity, just before PG injection; pre-injection, just before DMSO or WS-3 injection; and post-injection, just before the end of the latest injection. The changes in the low beta band power in ECoG were reported to be a good indicator of the degree of focal neocortical seizure (Ludvig et al., 2009) and suppression of EDs by FBC (Kida et al., 2012).

### Statistical Analysis

Statistical analyses were performed using StatMate version Ⅲ software (Advanced Technology for Medicine and Science, Chiba, Japan). All results are expressed as mean ± standard error of the mean (SEM). Statistically significant differences were evaluated by Tukey’s test, Dunnett’s test, Student’s *t*-test, or Welch’s *t*-test with *p* < 0.05 indicating statistical significance.

## Results

### Epileptic Seizures in TRPM8KO Mice Were Exacerbated by a Single High Dose of PTZ

We first investigated whether a lack of TRPM8 channels results in deteriorated epileptic seizures. Wildtype (WT) mice intraperitoneally administered a single dose of PTZ at 40 mg/kg exhibited ear and facial twitching, turning over onto the side, myoclonic jerks and rearing, and wild running (stages 1, 3, or 4). Administration of PTZ (40 mg/kg) to TRPM8KO mice induced generalized turning over onto the side, wild running and jumping, tonic-clonic seizures, or lethal seizures (stages 4, 5, or 6). PTZ administration resulted in a greater increase in seizure score in TRPM8KO mice compared with that in WT mice (*p* < 0.001, Student’s *t*-test; Figure 1).

### Suppressive Effects of TRPM8 Agonist on EDs in WT Mice

Icilin, a TRPM8 and TRPA1 agonist, suppressed PG-induced EDs (Moriyama et al., 2019), but it remains unknown whether selective TRPM8 agonists suppress EDs. Therefore, we investigated the effects of WS-3, a selective TRPM8 agonist, on PG-induced EDs. We continuously recorded ECoGs for 150 min in mice anesthetized with urethane (Figure 2A). Mice were injected with PG 30 min from the start of ECoG recording and were injected with vehicle or WS-3 90 min after PG injection (Figure 2A). EDs plateaued 90 min after PG injection (Figures 2B–G). The higher the dose of WS-3 (0.1–1 μM), the greater the amplitude of EDs was decreased in WT mice (Figures 2B–E). DMSO or low and middle doses of WS-3 did not affect the power of the low beta band in WT mice, whereas a high dose of WS-3 (1.0 mM) significantly suppressed the power of the low beta band (*p* = 0.042 vs. pre-injection group, Student’s *t*-test; Figure 2H).

### EDs Were Exacerbated by Lack of TRPM8 Channels

To elucidate the effects of a lack of TRPM8 channels on EDs, we compared EDs in WT and TRPM8KO mice. A lack of TRPM8 channels increased the intensity of low beta band power during pre-injection by approximately double compared with WT mice at the same dose of DMSO or WS-3 (*p* < 0.034 and *p* < 0.031, respectively, Welch’s *t*-test; Figure 2H). Administration of vehicle to TRPM8KO mice did not affect the PG-induced EDs, and this was not suppressed by 1.0 mM WS-3 (Figures 2F–H). In WT mice, EDs were focally induced by epilepsy-inducer, and were rarely propagated in the contralateral side (Figures 2B–E, *p* = 0.035, *p* = 0.064, respectively, Welch’s *t*-test; Figure 2I), whereas the EDs were easily propagated in the contralateral side by the lack of TRPM8 channels (Figures 2F,G, *p* = 0.003, *p* = 0.015, respectively, Welch’s *t*-test; Figure 2I). The intensity of low beta band that was observed in the contralateral side in was more increased than that in WT mice (*p* = 0.018, *p* = 0.042, respectively, Welch’s *t*-test; Figure 2I). In addition, the lack of TRPM8 channels resulted in a shorter firing latency of EDs by the epilepsy inducer compared with that in WT mice (*p* < 0.001, *p* < 0.001, respectively, Welch’s *t*-test; Figure 2J).

### Suppressive Effects of TRPM8 Agonist on Development of EDs and Seizure Score in WT Mice

To reveal the effects of TRPM8 agonists on the development of EDs and epileptic seizures, mice were injected with WS-3 30 min before receiving an epilepsy-inducer injection (Figure 3A). In WT mice, PG induced EDs when 1% DMSO was injected before the PG injection, whereas EDs were rarely detected when WS-3 was injected before the PG injection (Figures 3B,C). DMSO administration before PG injection did not affect the increased intensity of the beta band, whereas WS-3 administration before PG injection suppressed the increased intensity of the beta band by PG (*p* = 0.038, Welch’s *t*-test; Figure 3F). Injection of vehicle before PG injection in WT mice promoted myoclonic jerks and rearing or turning over onto the side, wild running and jumping (stage 3 or 4). In contrast, WS-3 pre-injection of WT mice resulted in no response or ear and facial twitching or convulsive twitching axially through the body (stage 0 or 2). The seizure score was 3.40 ± 0.24 by PG, which was reduced by WS-3 pre-injection (*p* = 0.002, Student’s *t*-test; Figure 3G).

### Lack of TRPM8 Channels Exacerbated Epileptic Seizures

To elucidate the effects of TRPM8 channel absence on the development of EDs and epileptic seizures, we compared EDs and seizure scores between WT and TRPM8KO mice. In TRPM8KO mice, PG injection after 1% DMSO or WS-3 injection induced EDs (Figures 3D,E), increasing the intensity of the beta band compared with basal activity (*p* < 0.001 and *p* < 0.001, respectively, Tukey’s test; Figure 3F). A lack of TRPM8 channels increased the intensity of low beta band power during post-injection, compared with WT mice at the same dose of DMSO (*p* < 0.045, Welch’s *t*-test; Figure 2H). In addition, PG injection after 1% DMSO or WS-3 injection of TRPM8KO mice resulted in turning over onto the side, wild running, and wild jumping or generalized tonic-clonic seizures (stages 4 or 5). The seizure score was higher in TRPM8KO mice compared with WT mice following PG injection after 1% DMSO injection, whereas WS-3 injection before PG injection did not reduce the seizure score in TRPM8KO mice (*p* = 0.020 and *p* = 1, respectively, Student’s *t*-test; Figure 3G).

## Discussion

This study investigated changes in EDs and epileptic seizures in a model lacking TRPM8 channels, as well as the effects of TRPM8 agonist on EDs and epileptic seizures. Our study had three major findings. First, TRPM8KO mice had a shorter firing latency of EDs than WT mice following administration of an epileptic inducer, with the EDs being more easily propagated to the contralateral side. Second, TRPM8 agonist injection of the epileptic focus suppressed EDs and seizures in WT mice, whereas anti-epileptic effects were not observed even when TRPM8KO mice were injected with the TRPM8 agonist in the epileptic focus. Third, in WT mice, TRPM8 agonist administration before ED-inducer resulted in the development of fewer EDs and a lower epileptic seizure score. In contrast, these anti-epileptic effects were not observed in TRPM8KO mice, and EDs and epileptic seizures were more exacerbated by the epilepsy-inducer in TRPM8KO mice compared with WT mice.

To clarify the effects of TRPM8 channel absence on PG-induced epilepsy, we recorded ECoGs in TRPM8KO mice. In mice that lacked TRPM8 channels, the latency of the development of the first ED was shortened by an epileptic inducer and the power of EDs was exacerbated, while the EDs were more easily propagated to the contralateral side and the seizure score was deteriorated (Figures 2, 3). This deterioration in epilepsy resulting from a lack of TRPM8 channels may be due to the regulation of excitatory cells by TRPM8 activation (Janssens et al., 2016). Wrigley et al. (2009) previously reported that icilin, a TRPM8 and TRPA1 agonist, reduced the amplitude of primary afferent-evoked glutamatergic excitatory postsynaptic currents (EPSCs). In contrast, other reports showed that (−)-menthol, a TRPM8 and TRPA1 agonist, increased the miniature excitatory postsynaptic current frequency (Tsuzuki et al., 2004; Luo et al., 2019). These conflicting results about the effects of TRPM8 activation on excitatory neurons may be because of the difference in TRPM8 affinity and the effects of cooling compounds on TRPA1. Because icilin is a more selective TRPM8 agonist than (−)-menthol (Mckemy Dd and Julius, 2002), icilin activates TRPM8 with more potency and affects excitatory neurons more than (−)-menthol. In contrast, Xu et al. (2015) reported that activation of TRPA1 channels increases the spontaneous release of L-glutamate onto substantia gelatinosa neurons. On the basis of the above findings, our results suggest that EDs and epileptic seizures are exacerbated when the epileptic focus lacks TRPM8.

In the present study, selective TRPM8 activation by WS-3 was found to suppress PG-induced EDs and seizures in WT mice. These anti-epileptic-like effects are in line with a previous report demonstrating that TRPM8 activation suppressed PG-induced EDs (Moriyama et al., 2019). In addition, anti-epileptic effects were not detected even when the TRPM8 agonist was administered to the epileptic focus in TRPM8KO mice (Figure 2). These results were supported by those of Moriyama et al., who reported that the anti-epileptic-like effects were antagonized when TRPM8 antagonist was administered just before TRPM8 agonist injection (Moriyama et al., 2019). These findings suggest that TRPM8 activation by TRPM8 agonists suppressed epilepsy. Before the distribution of TRPM8 channels in the rodent brain was reported (Ordas et al., 2019), opinions varied as to the effects of TRPM8 activation on excitatory neurons. Pezzoli et al. (2014) reported that icilin, a TRPM8 and TRPA1 agonist, can modulate glutamatergic neurons in the brain through a TRP-independent pathway. In single neurons of the peripheral nerves, it was reported that a low concentration of icilin (3 μM/L) to activate TRPM8 produced a decrease in the amplitude of evoked EPSCs that were obtained by electrical stimulation of the single dorsal rootlet neuron (Wrigley et al., 2009). These differing results could be explained by the difference in the TRPM8 expression rate between the brain and the peripheral nerves. Wrigley et al. reported that superfusion of a low concentration of icilin (3 mmol·L^−1^) produced a decrease in the amplitude of evoked EPSCs in 23% of neurons (Wrigley et al., 2009). Furthermore, Pezzoli et al. (2014) reported that TRPM8 channels were not expressed in the somatosensory cortex. Following the finding that a few TRPM8 channels exist in the rodent somatosensory cortex (Ordas et al., 2019), it was revealed that PG-induced EDs were suppressed by TRPM8 activation (Moriyama et al., 2019). These reports suggest that TRPM8 activation suppresses EDs despite TRPM8 channels being present in very low numbers in the rodent somatosensory cortex (Ordas et al., 2019).

Icilin at a dose of 3.0 mM eliminates PG-induced EDs for several minutes (Moriyama et al., 2019); therefore, we investigated the effects of TRPM8 agonist on ED development. Our data demonstrated that EDs and epileptic seizures in WT mice were rarely observed following the administration of the selective TRPM8 agonist before ED-inducer injection (Figure 3C). In contrast, EDs and epileptic seizures in TRPM8KO mice were observed even when the selective TRPM8 agonist was administered before ED-inducer injection (Figure 3E). These results suggest that TRPM8 activation suppressed the development of EDs and epileptic seizures that were caused by epileptic inducer, but the mechanisms of these anti-epileptic effects are unclear. Because PG inhibits GABA_A_-R (Tsuda et al., 1994; Sugimoto et al., 2002), the number of activated excitatory neurons may be increased by PG injection. To obtain a deeper understanding of these antiepileptic mechanisms, further experiments examining whether TRPM8 activation modulates extracellular glutamate is required.

The effects of TRPM8 agonist on EDs and epileptic seizures were evaluated using WT and TRPM8KO mice. Our present data showed that EDs were suppressed by TRPM8 agonist administration after epilepsy-inducer injection. In addition, ED development and epileptic seizures were suppressed by TRPM8 agonist administration before the epilepsy-inducer injection. In contrast, in TRPM8KO mice, EDs and epileptic seizures were not suppressed by TRPM8 agonist and were more deteriorated by the epileptic inducer compared with that in WT mice. Thus, further studies are required to determine the mechanism of the anti-epileptic effects of TRPM8 agonist. In conclusion, TRPM8 agonist may be the basis for the development of new drug treatments for patients with focal epilepsy.

## Data Availability

The raw data supporting the conclusion of this article will be made available by the authors, without undue reservation.

## References

[B1] Bautista D. M. Siemens J. Glazer J. M. Tsuruda P. R. Basbaum A. I. Stucky C. L. (2007). The Menthol Receptor TRPM8 Is the Principal Detector of Environmental Cold. Nature 448, 204–208. 10.1038/nature05910 17538622

[B2] Behrendt H. J. Germann T. Gillen C. Hatt H. Jostock R. (2004). Characterization of the Mouse Cold-Menthol Receptor TRPM8 and Vanilloid Receptor Type-1 VR1 Using a Fluorometric Imaging Plate Reader (FLIPR) Assay. Br. J. Pharmacol. 141, 737–745. 10.1038/sj.bjp.0705652 14757700 PMC1574235

[B3] Dhaka A. Murray A. N. Mathur J. Earley T. J. Petrus M. J. Patapoutian A. (2007). TRPM8 Is Required for Cold Sensation in Mice. Neuron 54, 371–378. 10.1016/j.neuron.2007.02.024 17481391

[B4] Fujii M. Inoue T. Nomura S. Maruta Y. He Y. Koizumi H. (2012). Cooling of the Epileptic Focus Suppresses Seizures with Minimal Influence on Neurologic Functions. Epilepsia 53, 485–493. 10.1111/j.1528-1167.2011.03388.x 22292464

[B5] Golyala A. Kwan P. (2017). Drug Development for Refractory Epilepsy: The Past 25 Years and beyond. Seizure 44, 147–156. 10.1016/j.seizure.2016.11.022 28017578

[B6] Janssens A. Gees M. Toth B. I. Ghosh D. Mulier M. Vennekens R. (2016). Definition of Two Agonist Types at the Mammalian Cold-Activated Channel TRPM8. Elife 5, e17240. 10.7554/eLife.17240 27449282 PMC4985286

[B7] Kida H. Fujii M. Inoue T. He Y. Maruta Y. Nomura S. (2012). Focal Brain Cooling Terminates the Faster Frequency Components of Epileptic Discharges Induced by Penicillin G in Anesthetized Rats. Clin. Neurophysiol. 123, 1708–1713. 10.1016/j.clinph.2012.02.074 22459055

[B8] Kwan P. Brodie M. J. (2000). Early Identification of Refractory Epilepsy. N. Engl. J. Med. 342, 314–319. 10.1056/NEJM200002033420503 10660394

[B9] Luo Y. Sun W. Feng X. Ba X. Liu T. Guo J. (2019). (-)-menthol Increases Excitatory Transmission by Activating Both TRPM8 and TRPA1 Channels in Mouse Spinal Lamina II Layer. Biochem. Biophys. Res. Commun. 516, 825–830. 10.1016/j.bbrc.2019.06.135 31262448

[B10] McKemy D. D. Neuhausser W. M. Julius D. (2002). Identification of a Cold Receptor Reveals a General Role for TRP Channels in Thermosensation. Nature 416, 52–58. 10.1038/nature719 11882888

[B11] Mizoguchi H. Nakade J. Tachibana M. Ibi D. Someya E. Koike H. (2011). Matrix Metalloproteinase-9 Contributes to Kindled Seizure Development in Pentylenetetrazole-Treated Mice by Converting Pro-BDNF to Mature BDNF in the hippocampus. J. Neurosci. 31, 12963–12971. 10.1523/JNEUROSCI.3118-11.2011 21900575 PMC6623408

[B12] Moriyama H. Nomura S. Kida H. Inoue T. Imoto H. Maruta Y. (2019). Suppressive Effects of Cooling Compounds Icilin on Penicillin G-Induced Epileptiform Discharges in Anesthetized Rats. Front. Pharmacol. 10, 652. 10.3389/fphar.2019.00652 31263415 PMC6585232

[B13] Nomura S. Fujii M. Inoue T. He Y. Maruta Y. Koizumi H. (2014). Changes in Glutamate Concentration, Glucose Metabolism, and Cerebral Blood Flow during Focal Brain Cooling of the Epileptogenic Cortex in Humans. Epilepsia 55, 770–776. 10.1111/epi.12600 24779587

[B14] Nomura S. Inoue T. Imoto H. Suehiro E. Maruta Y. Hirayama Y. (2017). Effects of Focal Brain Cooling on Extracellular Concentrations of Neurotransmitters in Patients with Epilepsy. Epilepsia 58, 627–634. 10.1111/epi.13704 28225164

[B15] Ordás P. Hernández-Ortego P. Vara H. Fernández-Peña C. Reimúndez A. Morenilla-Palao C. (2019). Expression of the Cold Thermoreceptor TRPM8 in Rodent Brain Thermoregulatory Circuits. J. Comp. Neurol. 529, 234–256. 10.1002/cne.24694 30942489

[B16] Pezzoli M. Elhamdani A. Camacho S. Meystre J. González S. M. Le Coutre J. (2014). Dampened Neural Activity and Abolition of Epileptic-like Activity in Cortical Slices by Active Ingredients of Spices. Sci. Rep. 4, 6825. 10.1038/srep06825 25359561 PMC4215320

[B17] Sheng J. Liu S. Qin H. Li B. Zhang X. (2018). Drug-Resistant Epilepsy and Surgery. Curr. Neuropharmacol 16, 17–28. 10.2174/1570159X15666170504123316 28474565 PMC5771378

[B18] Sugimoto M. Fukami S. Kayakiri H. Yamazaki S. Matsuoka N. Uchida I. (2002). The Beta-Lactam Antibiotics, Penicillin-G and Cefoselis Have Different Mechanisms and Sites of Action at GABA(A) Receptors. Br. J. Pharmacol. 135, 427–432. 10.1038/sj.bjp.0704496 11815378 PMC1573156

[B19] Tsuda A. Ito M. Kishi K. Shiraishi H. Tsuda H. Mori C. (1994). Effect of Penicillin on GABA-Gated Chloride Ion Influx. Neurochem. Res. 19, 1–4. 10.1007/BF00966719 8139755

[B20] Tsuzuki K. Xing H. Ling J. Gu J. G. (2004). Menthol-induced Ca2+ Release from Presynaptic Ca2+ Stores Potentiates Sensory Synaptic Transmission. J. Neurosci. 24, 762–771. 10.1523/JNEUROSCI.4658-03.2004 14736862 PMC6729265

[B21] Wrigley P. J. Jeong H. J. Vaughan C. W. (2009). Primary Afferents with TRPM8 and TRPA1 Profiles Target Distinct Subpopulations of Rat Superficial Dorsal Horn Neurones. Br. J. Pharmacol. 157, 371–380. 10.1111/j.1476-5381.2009.00167.x 19371346 PMC2707984

[B22] Xu Z. H. Wang C. Fujita T. Jiang C. Y. Kumamoto E. (2015). Action of Thymol on Spontaneous Excitatory Transmission in Adult Rat Spinal Substantia Gelatinosa Neurons. Neurosci. Lett. 606, 94–99. 10.1016/j.neulet.2015.08.042 26314510

